# Genome-Wide Detection of Selection Signatures in Duroc Revealed Candidate Genes Relating to Growth and Meat Quality

**DOI:** 10.1534/g3.120.401628

**Published:** 2020-08-28

**Authors:** Jian Yu, Pengju Zhao, Xianrui Zheng, Lei Zhou, Chuduan Wang, Jian-Feng Liu

**Affiliations:** National Engineering Laboratory for Animal Breeding; Key Laboratory of Animal Genetics, Breeding, and Reproduction, Ministry of Agriculture; College of Animal Science and Technology, China Agricultural University, Beijing, 100193, China

**Keywords:** Whole-genome sequencing, Duroc pig, Selection signatures

## Abstract

With the development of high-throughput genotyping techniques, selection signatures in the genome of domestic pigs have been extensively interrogated in the last decade. The Duroc, a major commercial pig breed famous for its fast growth rate and high lean ratio, has not been extensively studied focusing on footprints of intensively artificial selection in their genomes by a lot of re-sequencing data. The goal of this study was to investigate genomic regions under artificial selection and their contribution to the unique phenotypic traits of the Duroc using whole-genome resequencing data from 97 pigs. Three complementary methods (d_i_, CLR, and iHH12) were implemented for selection signature detection. In Total, 464 significant candidate regions were identified, which covered 46.4 Mb of the pig genome. Within the identified regions, 709 genes were annotated, including 600 candidate protein-coding genes (486 functionally annotated genes) and 109 lncRNA genes. Genes undergoing selective pressure were significantly enriched in the insulin resistance signaling pathway, which may partly explain the difference between the Duroc and other breeds in terms of growth rate. The selection signatures identified in the Duroc population demonstrated positive pressures on a set of important genes with potential functions that are involved in many biological processes. The results provide new insights into the genetic mechanisms of fast growth rate and high lean mass, and further facilitate follow-up studies on functional genes that contribute to the Duroc’s excellent phenotypic traits.

Pigs (*Sus scrofa*) play an important role economically, supplying energy and abundant animal protein for humans. In addition, they also can serve as an ideal animal model for research on human diseases, because they have similar physiological characteristics to humans (Lunney 2007). Pigs were domesticated in Europe and Asia approximately 9000 years ago from the Eurasian wild boar (Larson *et al.* 2005). Since then, the pig has experienced natural and artificial selection to meet the increased human demand for food, which has reduced genetic diversity and increased linkage disequilibrium in selected regions (Ai *et al.* 2013; Akey *et al.* 2010). Intense artificial selection has led to different pig breeds with unique, distinct phenotypic and physiological features (Ai *et al.* 2015; Bosse *et al.* 2012; Frantz *et al.* 2015). With the development of genome-wide genotyping and sequencing technology, researchers can readily detect variations in genomic distribution and use state-of-the-art statistical methods to detect selection signatures in order to explore the genetic mechanisms underlying the unique Duroc phenotype caused by selection (Mokhber *et al.* 2018; Moon *et al.* 2015).

There are at least 16 species in the Suidae family around the world, and most of them are distributed in Europe and Asia (Giuffra *et al.* 2000; Li *et al.* 2012). Of the pig breeds, the Duroc is a well-known domesticated pig breed because of its superior performance in growth, carcass, and feed conversion efficiency due to intense artificial selection for more than 100 years ago (Cameron and Enser 1991). Therefore, the Duroc is often used as the terminal sires, *i.e.*, used as parental sires in the three-way crossbreeding system, to improve the lean percentage and growth rate of hybrid pigs in pig industry. These selection processes have left a unique footprint in genomic regions causing the excellent production traits of the Duroc. So far, a few studies have focused on the detection of selection signatures in the Duroc genome, and there are limited indications of potential functional genes for its superior growth and carcass characteristics (Edea *et al.* 2017; Yang *et al.* 2014).

We suspected that the difference in phenotype between Duroc and other pig breeds may be due to different selection pressure in the Duroc genome, which causing genetic mutation in some important genes associated with growth and carcass quality. Detecting the mutations would facilitate future breeding of Durocs to improve these traits through genomic selection. Consequently, large-scale resequencing was performed in 31 unrelated Duroc and other domesticated pigs to detect genomic evidence for the excellent productive phenotype of the Duroc and to identify promising genes for future application in pig breeding.

## Materials and Methods

### Sample collection and sequencing

In this study, 63 samples were collected from different areas in China, which included 32 unrelated Meishan samples from Kunshan, Jiangsu Province, and 31 unrelated Duroc samples from Yancheng, Jiangsu Province. DNA was extracted from ear tissue using the Qiagen DNeasy Tissue kit (Qiagen, Germany), and then analyzed by spectrophotometry and agarose gel electrophoresis to ensure high quality. Subsequently, all samples were constructed from a library (paired-end, 2 × 125 bp) and sequenced using an Illumina HiSeq 2000 sequencing system at Novogene (Beijing, China). Finally, a total of 1,403.35 Gb of sequence data were generated, which were used for subsequent analysis. In addition, genomic data of 4 Duroc and 30 Tibetan wild boars (Li *et al.* 2013; Zhao *et al.* 2016) were downloaded from NCBI with accession numbers ERP001813 and SRA065461, respectively. In total, there was whole-genome sequencing data for 32 Meishan samples, 35 Duroc samples, and 30 Tibetan pig samples in this study.

### Reads quality control and mapping

For raw reads, three quality control criteria were checked to ensure high accuracy in reads mapping using the NGSQC Toolkit (Patel and Jain 2012): First, trimming reads contained adapter sequences; second, undesirable reads contained no less than 30% low-quality bases were discarded (quality value ≤ 20, or N bases); and third, low-quality 3ˈ end reads whose base quality score was lower than 20 were modified. Subsequently, the clean reads (including all the autosomes and the X chromosome) were aligned to the swine reference genome (*Sus scrofa* 11.1ftp://ftp.ensembl.org//pub/release-99/fasta/sus_scrofa/dna/) using Burrows-Wheeler Aligner (BWA) (Li and Durbin 2009) with default parameters. Duplicated reads were removed using Picard tools (http://picard.sourceforge.net). In addition, base quality recalibration and mapping statistics (such as sequence depth) were implemented for subsequent analyses using Genome Analysis Toolkit (GATK) (McKenna *et al.* 2010) and SAMtools (Li *et al.* 2009) respectively.

### Single nucleotide variants (SNVs) calling and annotation

After alignment, SAMtools was then used to convert SAM into the best alignments in BAM format. The ‘SortSam’, ‘MarkDuplicates’, ‘BaseRecalibrator’ of GATK were used separately to sorting, to remove potential PCR duplications and for base quality score recalibrations. The BAM files were indexed by SAMtools. The ‘HaplotypeCaller’, ‘GenomicsDBImport’ and ‘GenotypeGVCFs’ of GATK with default parameters were used to call SNPs, which generated genotype calls in Variant Call Format (VCF). We further removed those INDELs in the VCF file using ‘SelectVariants’ of GATK. Finally, the GATK ‘VariantFiltration’ module was used to exclude SNP calling errors in according to the following criteria (McKenna *et al.* 2010). High-quality SNPs with Quality score > 30, MQ RMS mapping quality >20, DP > 5, coverage > 30%, and MAF >0.01 were kept for further analysis. In addition, the dbSNP database (Sherry *et al.* 2001) was used to identify novel genetic variations. Finally, All filtered SNPs were functionally annotated using the gene-based annotation modules of ANNOVAR software (Wang *et al.* 2010), and the corresponding gene annotation file was downloaded from the Ensembl database (https://hgdownload.soe.ucsc.edu/goldenPath/susScr11/bigZips/genes/susScr11.ensGene.gtf.gz). In the annotation step, SNVs were classified into eight categories based on their genome locations, including exonic regions (synonymous, nonsynonymous, stop gain and stop loss), splicing sites, intronic regions, 5ˈ and 3ˈ untranslated regions (UTRs), upstream and downstream regions, and intergenic regions.

### Selection signature detection of the Duroc population

To detect the genomic region under selection in the Duroc, three complementary methods were implemented to ascertain the selection signatures. First, the population differentiation coefficient (F_ST_) (Holsinger and Weir 2009) was used to measure population differentiation between the Duroc and the other two populations (Meishan and Tibetan wild boars) using non-overlapping 100kb windows. The F_ST_ was further translated into the d_i_ statistic (Akey *et al.* 2010) for each SNP, *i.e.*, $d_{i}=\underset{i\neq j}{\sum }\frac{F_{ST}^{ij}-E[F_{ST}^{ij}]}{sd[F_{ST}^{ij}]}$, where $E[F_{ST}^{ij}]$ and $sd[F_{ST}^{ij}]$ represent the expected value and standard deviation of F_ST_ between breeds i (Duroc) and j (Meishan or Tibetan wild boars) calculated from 18 autosomes. The d_i_ values for Duroc *vs.* Meishan and Duroc *vs.* Tibetan for each locus were summed, and the d_i_ values were averaged over SNVs in non-overlapping 100-kb windows. We empirically selected extremely high d_i_ values in the 5% right-tail as potential candidate regions under positive selection.

Second, a recently reported method, Integrated Haplotype Homozygosity Pooled (iHH12)(Torres *et al.* 2018), was used to detect selection signatures in the Duroc genome. Beagle software (Browning and Browning 2007) was used to impute and phase the missing genotypes of the Duroc (with default settings) before iHH12 was calculated. The iHH12 method was developed based on a window-based statistic (H12) (Garud *et al.* 2015) with good power to detect soft sweeps by adapting H12 into an integrated haplotype homozygosity framework. For this statistic, EHH12 of the entire sample of haplotypes from different loci was first calculated. Based on that, iHH12 was calculated. And We calculated iHH12 values for each loci using Selscan software (Szpiech and Hernandez 2014). Finally, the iHH12 values are normalized across the whole genome, *i.e.*, $iHH12=\frac{iHH12-E[iHH12]}{sd[iHH12]}$. The genome was divided into non-overlapping 100kb windows. We empirically selected extremely high iHH12 values in the 5% right-tail as potential candidate regions under positive selection.

Lastly, the composite likelihood ratio (CLR) test (Pavlidis *et al.* 2013) was implemented to search for a recent selection sweep caused by a change in allele frequency at linked sites, which generates an excess of derived alleles at high frequencies and reducing genetic diversity (Fay and Wu 2000; Kim and Stephan 2002). CLR values were calculated for sites every 1kb across the genome using SweepFinder2 software (DeGiorgio *et al.* 2016), and the genome was divided into non-overlapping 100kb windows. We empirically selected extremely high CLR values in the 5% right-tail as potential candidate regions under positive selection. To further control the false positive rates of the detection of selection signatures, genomic regions identified by at least two methods were used in further analyses.

### Gene annotation and enrichment analysis

All identified candidate regions were annotated to further identify potential candidate genes based on the Ensembl Genes 100 Database (http://asia.ensembl.org/). Subsequently, these candidate genes were transformed into homologous identical genes in the human genome using the BioMart database (http://asia.ensembl.org/biomart/martview/) (Durinck *et al.* 2005), due to incomplete gene function annotations in the pig genome. We further investigated the function of annotated genes undergoing positive selection by Gene Ontology (GO) term analysis and KEGG pathway analysis using KOBAS (Wu *et al.* 2006) to reveal the potential biological function of candidate genes harbored in selected regions. Furthermore, we downloaded the published pig QTL from the Pig QTLdb (http://www.animalgenome.org/cgi-bin/QTLdb/SS/download?file=bedSS_11.1) to identify QTL overlapping with candidate selected regions in the current study based on the putative QTL.

### Data availability

A total 63 pig samples with 1,403.35Gbases were uploaded to NCBI with BioProject ID: PRJNA378496. Illumina paired-end sequences for the other 34 pigs used in this study were downloaded from NCBI with accession numbers ERP001813 and SRA065461. Supplemental material available at figshare: https://doi.org/10.25387/g3.11944890.

## Results

### Whole-genomic resequencing and SNV detection

**Figure 1:**
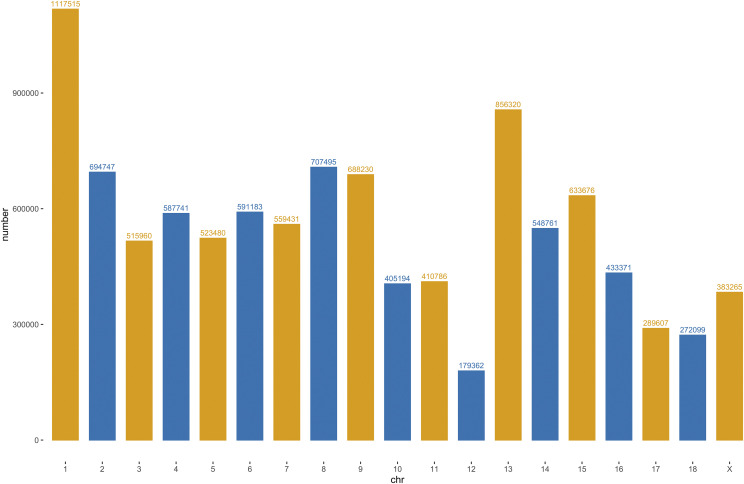
Genome-wide distribution of detected SNVs on 19 chromosomes for the Duroc. *X*-axis represents 18 autosomes and X chromosome. *Y*-axis represents the number of SNVs.

Whole-genome resequencing of 63 samples generated 1,403.35 G raw paired-end reads. After quality control, the clean reads were aligned to the *sus scrofa* 11.1 reference genome (ftp://ftp.ensembl.org//pub/release-99/fasta/sus_scrofa/dna/) using BWA. The sequence depth for each sample ranged from 3.92X to 13.43X, with an average of > 9.35X (Table S1). Subsequently, we called SNVs on a population level. Finally, 37,438,998 SNVs were we identified in Duroc genome. After filtration of the raw variants, 10,398,223 high-quality SNVs were kept for subsequent analyses. Approximately 87% of the variants (8,969,913 SNVs) were found in the dbSNP database, whereas more than 13% (1,428,310 SNVs) of the variants that we identified were absent from the SNPs database. These novel SNVs substantially expand the database of Duroc genetic variants. Genome-wide distribution of detected SNVs for Duroc were showed in Figure 1.

**Figure 2:**
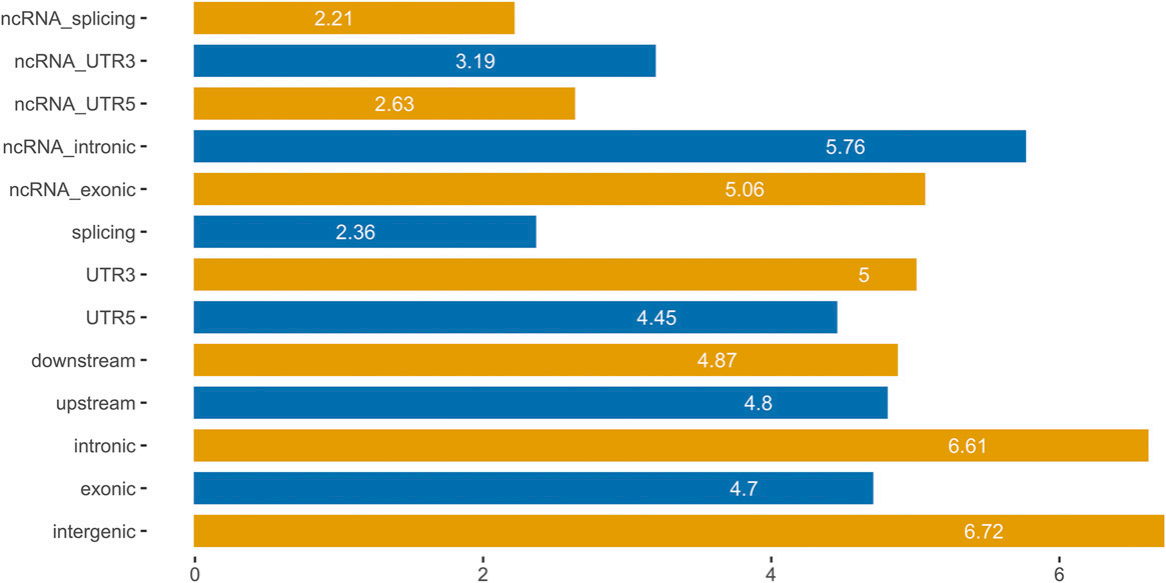
Genome-wide annotation of genetic variations. *X*-axis represents the number of genetic variations (log10) within various functional regions. *Y*-axis represents various functional regions in genes.

All detected SNVs in the Duroc population were annotated using a gene annotation file(https://hgdownload.soe.ucsc.edu/goldenPath/susScr11/bigZips/genes/susScr11.ensGene.gtf.gz) downloaded from the Ensembl database. Most of the SNVs were located in intergenic regions (∼51.06%), followed by introns (∼44.76%), upstream and downstream regions (∼1.32%), exons (∼1.59%), untranslated regions (UTRs) (∼1.24%), and splicing site regions (∼0.004%) (Figure 2). Further research on exonic regions detected 15,198 synonymous mutations, 14,177 non-synonymous mutations, 303 stop-gain mutations, and 49 stop-loss mutations. In addition, the results of functional annotation were used to identify the causal mutants in these candidate genes. Studying the functional variations provides an opportunity to explain the genetic mechanism of the features of the Duroc due to artificial selection.

### Detection of selection signatures

**Figure 3:**
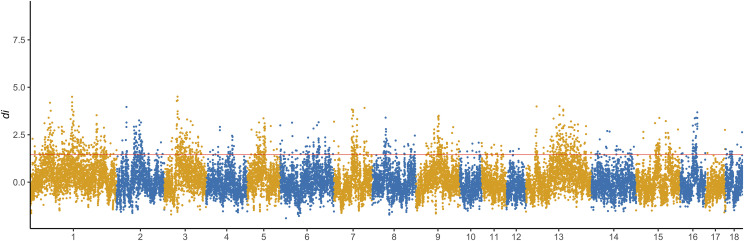
Genome-wide distribution of selection signatures detected by d_i_ on 18 chromosomes. *X*-axis represents 18 autosomes, and *Y*-axis represents d_i_ values for non-overlapping 100 kb windows. Red line displays the threshold level of 5%.

Our previous study showed that strong linkage disequilibrium (LD) levels in the Duroc compared to the other breeds because of intense artificial selection (Zhao *et al.* 2018). Based on these findings, it was speculated that there should be specific selection signatures in the Duroc population arising from long-term artificial selection after domestication. Therefore, positive selection signature detection was performed on the genome of Duroc pigs with large-scale resequencing data combining the three complementary methods with non-overlapping windows. Across the genome, d_i_ values for Duroc to Meishan and Tibetan wild boar populations with non-overlapping 100-kb windows ranged from -1.89 to 4.51, with an average of 0.087. The genome-wide distribution of the averaged d_i_ values for 100-kb non-overlapping windows is shown in Figure 3. Extremely high di values (d_i_ > 1.45) located at the 5% right tail of the empirical distribution were selected as potential candidate regions of artificial selection in the genome of the Duroc.

**Figure 4:**
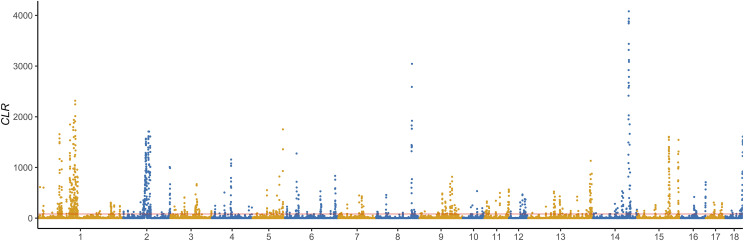
Genome-wide distribution of selection signatures detected by CLR on 18 chromosomes. *X*-axis represents 18 autosomes, and *Y*-axis represents CLR values for non-overlapping 100 kb windows. Red line displays the threshold level of 5%.

**Figure 5:**
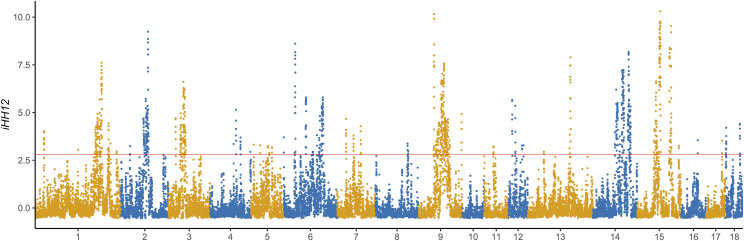
Genome-wide distribution of selection signatures detected by iHH12 on 18 chromosomes. *X*-axis represents 18 autosomes, and *Y*-axis represents iHH12 values for non-overlapping 100 kb windows. Red line displays the threshold level of 5%.

Furthermore, two within-population methods (CLR and iHH12) were implemented to detect the selection signatures of Duroc. The genome-wide distribution of CLR values for 100-kb non-overlapping windows is shown in Figure 4. The CLR values for these windows ranged from ∼0 to 4,080, with an average of 28.27. Extremely high CLR values (CLR > 81.85) at the 5% right tail of the empirical distribution were selected as potential candidate signals in the Duroc. For iHH12 detection, extremely high iHH12 values (iHH12 > 2.81) at the 5% right tail of the empirical distribution were selected as potential candidate signals for positive selection in the Duroc. Accordingly, the iHH12 values for windows ranged from 0 to 10.31, with an average value of 0.27. The iHH12 further confirmed some strong selection signatures on SCC6, SSC14, and SSC15, which were identified in CLR (Figure 5).

**Figure 6:**
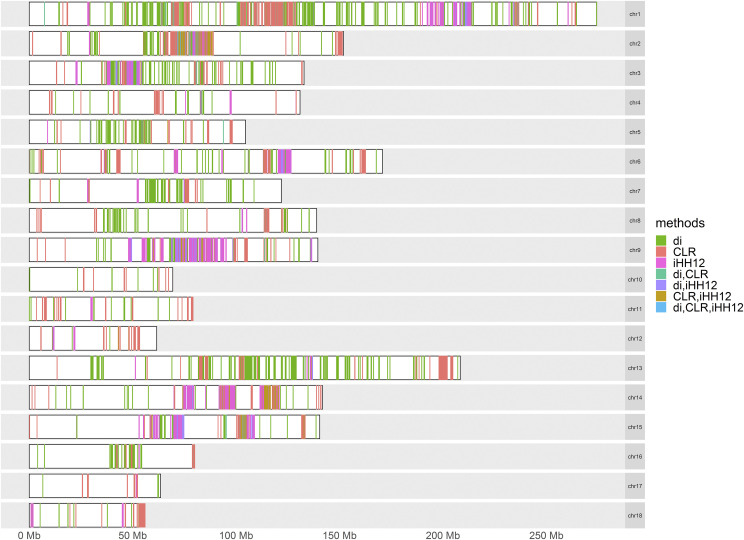
Genomic distribution of selection signatures detected by d_i_, CLR and iHH12 on 18 chromosomes. In the figure, “d_i_”,” CLR” and “iHH12” represent the regions detected by only one method; “d_i_,CLR”, “d_i_,iHH12” and “CLR,iHH12” represent the regions detected by two different methods; “d_i_,CLR,iHH12” represents the regions detected by all three methods.

**Figure 7:**
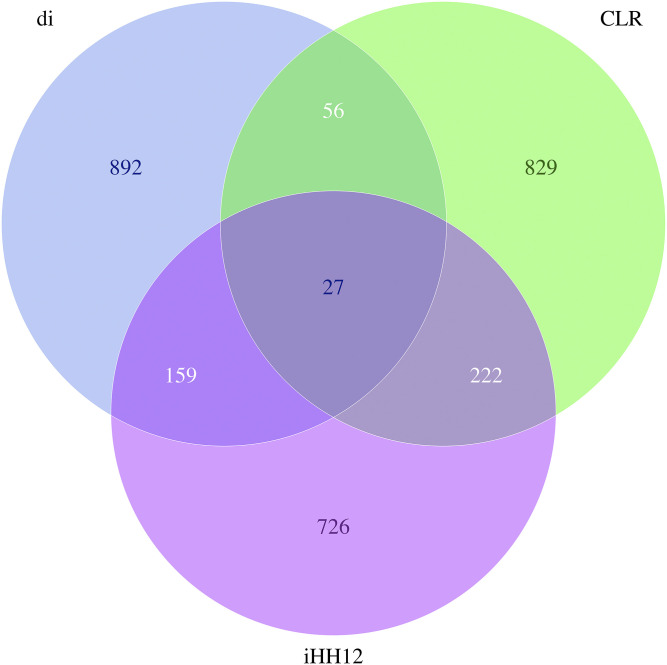
Venn diagram for numbers of candidate regions detected by different methods. Blue represents the numbers of candidate regions detected by d_i_; green represents the numbers of candidate regions detected by CLR; orchid represents the numbers of candidate regions detected by iHH12.

In order to reduce false positive candidate regions, potential candidate regions identified by more than two methods were chosen as the final candidate regions for positive selection. The genome distribution of candidate regions detected by different methods is shown in Figure 6. A total of 464 candidate regions (Figure 7) were identified by more than two methods, which were mainly distributed on SSC1, SSC2, and SSC9, covering 46.4 Mb genomic regions. Meanwhile, 27 candidate regions were identified by all three methods.

### Gene annotation and functional analysis

All identified candidate regions were annotated to identify functionally important genes relevant to the excellent phenotype of the Duroc, such as high daily gain, meat content in the carcass and feed conversion ratio. Seven hundred and nine genes were identified in candidate regions, including 600 candidate protein code genes (486 functionally annotated genes) (Table S6) and 109 lncRNA genes (Table S7). There were many genes involved in important biological processes, such as *insulin receptor* (*INSR*), *insulin-like growth factor 1 receptor (IGF1R)*, and *insulin-like growth factor 2 receptor (IGF2R)* for muscle growth and protein synthesis. Further analysis of these genes revealed that the *INSR* gene (identified by all three methods) has underwent intense selection (d_i_ = 3.25, CLR = 935.59, iHH12 = 4.68). Functional annotation showed that the *INSR* gene contains 114 SNVs, most of which are intronic variations. In particular, a synonymous SNV (ENSSSCG00000013566:ENSSSCT00000014817:exon12:c.T2640C:p.G880G; ENSSSCG00000013566:ENSSSCT00000014815:exon13:c.T2676C:p.G892G; rs338663742 in the dbSNP database) was found within the *INSR* gene. Intriguingly,this mutation was significantly different between the Duroc with Meishan and Tibetan populations (Table S8).

The functions of these annotated genes undergoing positive selection was investigated further by GO term analysis and KEGG pathway analysis using KOBAS. Considering insufficiency in pig genome gene function annotations, the candidate genes were transformed into homologous identical genes in the human genome using the BioMart database (http://asia.ensembl.org/biomart/martview/). Finally, a total of 271 significantly enriched GO terms with P-values < 0.05 were observed (Table S9) that were associated with growth, protein synthesis, and metabolism, such as insulin-like growth factor receptor signaling pathway (GO:0048009), protein modification process (GO:0036211), regulation of biological process (GO:0050789), and regulation of protein metabolic process (GO:0051246). In addition, some of these significantly enriched terms were involved in energy metabolism and the immune response.

In addition, 53 significantly enriched KEGG pathway terms were observed (Table S10), including regulation of actin cytoskeleton (hsa04810), insulin resistance signaling pathway (hsa04931), and PI3K-Akt signaling pathway (hsa04151). Seven genes (*INSR*, *PDPK1*, *OGA*, *GFPT2*, *CREB3*, *CREB3L3*, and *PPP1R3E*) were significantly enriched in the insulin resistance signaling pathway, which plays an important role in energy metabolism, protein synthesis, and metabolism in muscle cells. Previous studies have reported that knockdown of *CREB3* in mice results in decreased estradiol and progesterone synthesis and promotes cell proliferation (Zhao *et al.* 2016).

The Pig QTLdb database (Release 41, Apr 26, 2020), which contain 30,580 QTL of 691 different traits reported in the past decades, was downloaded to identify quantitative trait loci (QTL) overlapping with candidate selected regions. In total, 3,404 porcine QTL (Table S11) were identified to be located within or overlapping with the candidate regions. Of these, 2,665 (∼78.29%) were associated with production and carcass traits, suggesting selection for production traits during the breeding of Duroc pigs.

## Discussion

Natural and artificial selection has left distinct imprints in the genomes of domestic animals, such as reduced genetic diversity and increased linkage disequilibrium in regions under selection (Ai, Huang, and Ren 2013; Akey *et al.* 2010). Identifying these regions in domesticated animals can help identify causal variants contributing to economic traits. In the current study, a number of regions with signatures of positive selection were identified, and further functional analyses on these regions identified a list of genes involved in fast growth and high lean deposition, supporting that these regions have been under selection. These findings begin to elucidate the genetic mechanism of the excellent phenotypic traits of the Duroc.

Previous studies have shown that it is more powerful to utilize complementation of different methods to detect selection signatures for livestock and poultry (Ma *et al.* 2015a; Qanbari *et al.* 2014). Therefore, three methods based on different sources of information were used to construct a comprehensive selection map for the Duroc genome in our study. Regions identified by at least two methods were identified as candidate regions of positive selection to reduce false positives. Although some older selection signatures could have been lost in some way, it does make the results more reliable, which was well supported by the QTL identified as overlapping with candidate selected regions. Most QTL (∼78.29%) were associated with production and carcass traits. In addition, most of the regions identified by all three methods had an extremely high signal value in the three methods, whereas the regions identified by only two methods tended to have a high signal value in one particular method. This means that regions identified by only two methods may have higher false positive rates than regions identified by all three methods. Previous study also suggested that combining different selection signature statistics can reduce those selective sweeps merely derived from independent domestication processes (Ma *et al.* 2015a). Compared with previous genome-wide scans for selection signatures in Duroc pigs (Edea *et al.* 2017; Diao *et al.* 2018; Ma *et al.* 2018), a number of overlapping signatures were found with previous studies, such as 7.3–7.4 Mb on SSC1, and 88.1–88.4 Mb on SSC2. These regions contain some important genes, such as *IGF2R*, *HOMER1* and *CMYA5*, which are associated with muscle development, carcass traits and meat quality (Hao *et al.* 2017; Xu *et al.* 2011). Some novel candidate regions containing genes associated with growth and carcass quality, such as *INSR* and *IGF1R*, were also found in the current study. These findings may have led to a gap in detecting selective sweeps in Duroc pigs.

Compared with the Duroc, the Meishan and Tibetan wild boar have an extremely slow growth rate and low lean meat ratio because of various breeding goals during long-term domestication, which has made a huge difference in their genomes. Therefore, this research design using Meishan and Tibetan wild pigs as reference populations has facilitated the accurate identification of genetic mechanisms under great phenotypic difference. Of the candidate genes, *INSR*, *IGF1R*, and *IGF2R*, are of particular significance, as they are synonymous with growth regulation and protein metabolism also seen in the Duroc. In particular, a synonymous exonic SNV within *INSR* gene was found, which was identified by all three methods with extremely high signal values. In the meanwhile, a novel heterozygous nonsense mutation in exon 2 of *INSR* was found in humans in a previous report (Saito-Hakoda *et al.* 2018), which causes type A insulin resistance. Interestingly, all Duroc pigs were homozygous wild type genotypes, and heterozygous mutations existed only in the Meishan and Tibetan pig populations. Therefore, it is suspected that the *INSR* gene may be a positional candidate gene explaining why the Duroc has excellent production traits. Moreover, *IGF1R* and *IGF2R* also play a crucial role in regulating cell proliferation and muscle growth. Previous studies have shown that *IGF1R* affects muscle growth and protein turnover (Schiaffino and Mammucari 2011; Meek *et al.* 1998) by regulating insulin and *IGF-1* to stimulate muscle protein synthesis (Fulks *et al.* 1975; Rommel *et al.* 2001) and inhibit protein degradation via the ubiquitin-proteasome and autophagy-lysosome pathways (Mammucari *et al.* 2007; Sandri *et al.* 2004). It is notable that *IGF2R* has previously been implicated in distinct patterns of selection in domesticated Duroc pigs and Tibetan wild boars (Li *et al.* 2013) and as a possible positional candidate gene detected by F_ST_ and CLR when comparing Duroc pigs with Asian wild boars and European wild boars (Ma *et al.* 2018). It has been reported that a new quantitative trait nucleotide is located in the regulatory sequence of the imprinted *IGF-2* gene, which explains 15–30% of the phenotypic variation in muscle mass and 10–20% of the variation in back-fat thickness (Van Laere *et al.* 2003). Therefore, *INSR*, *IGF1R* and *IGF2R* may be possible positional candidate genes explaining why Duroc pigs have excellent production traits.

Enrichment analysis showed that some important genes were significantly enriched in insulin resistance, which is a common pathophysiological mechanism of obesity and type 2 diabetes mellitus in humans (DeFronzo 1997; DeFronzo *et al.* 1985). Skeletal muscle is one of the major target organs of insulin-mediated glucose uptake, metabolism and utilization, and it is the earliest and most important site of insulin resistance. Recent studies have reported that the impairment of glucose uptake, insulin signaling pathway and mitochondrial biosynthesis are closely related to skeletal muscle insulin resistance (Asmann *et al.* 2006; Chavez-Velazquez *et al.* 2007; Toledo *et al.* 2006; Abdul-Ghani *et al.* 2008). Seven candidate genes (*INSR*, *PDPK1*, *OGA*, *GFPT2*, *CREB3*, *CREB3L3*, and *PPP1R3E*) are involved in this biological process, which is closely related to cell proliferation and metabolism. These candidate genes can elucidate domestication history in Duroc populations, and potentially offer new ways to explore evolutionary mechanisms.

In addition to these findings associated with growth and carcass quality, some important functional genes associated with immunity and transcription (such as *PALM*, *PTBP1*, and *CRHBP*) are also detected under selection pressure between European pigs and Asian pigs (Ma *et al.* 2015b; Moon *et al.* 2015), which may explain the difference in adaptability between Duroc and Asian pigs.

## Conclusion

In summary, we identified selection signatures in the genome of Duroc pig, a well-known European domestic pig breed. We found some promising genes associated with fast growth rate and high lean ratio of Duroc. These results provide novel insights into the genome evolution and selection mechanisms in domesticated pig breeds.
